# Enhancing Permeation of Drug Molecules Across the Skin via Delivery in Nanocarriers: Novel Strategies for Effective Transdermal Applications

**DOI:** 10.3389/fbioe.2021.646554

**Published:** 2021-03-29

**Authors:** Yi-Qun Yu, Xue Yang, Xiao-Fang Wu, Yi-Bin Fan

**Affiliations:** ^1^Scientific Research and Education Department, Chun’an First People’s Hospital (Zhejiang Provincial People’s Hospital Chun’an Branch), Hangzhou, China; ^2^Nursing Department, Chun’an First People’s Hospital (Zhejiang Provincial People’s Hospital Chun’an Branch), Hangzhou, China; ^3^Key Laboratory of Tumor Molecular Diagnosis and Individualized Medicine of Zhejiang Province, Zhejiang Provincial People’s Hospital, People’s Hospital of Hangzhou Medical College, Hangzhou, China; ^4^Department of Dermatology, Zhejiang Provincial People’s Hospital, People’s Hospital of Hangzhou Medical College, Hangzhou, China

**Keywords:** skin, transdermal, biomaterial, nanocarrier, nanotechnology

## Abstract

The transdermal route of administration provides numerous advantages over conventional routes i.e., oral or injectable for the treatment of different diseases and cosmetics applications. The skin also works as a reservoir, thus deliver the penetrated drug for more extended periods in a sustained manner. It reduces toxicity and local irritation due to multiple sites for absorption and owes the option of avoiding systemic side effects. However, the transdermal route of delivery for many drugs is limited since very few drugs can be delivered at a viable rate using this route. The stratum corneum of skin works as an effective barrier, limiting most drugs’ penetration posing difficulty to cross through the skin. Fortunately, some non-invasive methods can significantly enhance the penetration of drugs through this barrier. The use of nanocarriers for increasing the range of available drugs for the transdermal delivery has emerged as a valuable and exciting alternative. Both the lipophilic and hydrophilic drugs can be delivered via a range of nanocarriers through the stratum corneum with the possibility of having local or systemic effects to treat various diseases. In this review, the skin structure and major obstacle for transdermal drug delivery, different nanocarriers used for transdermal delivery, i.e., nanoparticles, ethosomes, dendrimers, liposomes, etc., have been discussed. Some recent examples of the combination of nanocarrier and physical methods, including iontophoresis, ultrasound, laser, and microneedles, have also been discussed for improving the therapeutic efficacy of transdermal drugs. Limitations and future perspectives of nanocarriers for transdermal drug delivery have been summarized at the end of this manuscript.

## Introduction

Oral administration is the typical route for drug delivery, but the drawbacks such as first-pass metabolism, high cost, and repeated dosing create this route challenging. A new drug delivery mechanism needs to be developed to enhance the therapeutic effectiveness, stabilization, and protection of medications to address all these challenges (Shaw and Mitchell, 1983). Stoughton has predicted the percutaneous absorption of drugs long ago in 1965, which tiled the technique for the topical/transdermal drug delivery system (TDDS) (Idson, 1975). Medications through TDDS offer an extended-spectrum for application to different shapes and sizes of formulations of single or more than one active constituents that, when applied on the integral skin, can distribute to all over the body or can act locally at the site of application. The wide-scale epidermis and informal functionality make it an ideal route to the penetration of drugs (Prausnitz and Langer, 2008). Though, the crucial trials in the formulation of TDD are prerequisites to address the very high impermeable, keratinized external layer skin, for example, ‘stratum corneum’ (SC) for successful translation to clinics. The core function of the stratum corneum is to give a protective layer for the tissues beneath the skin, however, this blockade function often decreases the quantity of drugs that can move through the skin passively (Prausnitz et al., 2004).

In nanoparticle technology, the range of particles is 1–500 nm to cure diseases and diagnose and has overcome the highly significant issue of low bioavailability of drugs. There have been many innovative studies and some of them are patented nanotechnology-based products in the medical field over the last few decades (Boisseau and Loubaton, 2011). The purpose of this technique is to identify and resolve issues of drugs for enhancing wellbeing by minimizing the adverse effects of drugs with non-invasive treatments. Nanoparticles deliver a lot of new resources to encounter these achievements. The alteration of drugs and other ingredients on a nanometer scale through nanotechnological approaches significantly enhances the therapeutic outcomes of these materials and reduces the associated side effects. Surface area, site-targeting, solubility enhancement, control delivery, and sustained release are a few properties that distinguish nanomedicines from conventional counterparts (Hughes, 2005). The field of nanocarriers based medicine is constantly growing and it has achieved success in curing cancers and infectious disorders that were hard to treat in the past (Díaz-Torres and Transdermal nanocarriers, 2010).

The use of such preparations on the skin for therapeutic reasons is as ancient as the medical history, analogous to the use of salves and ointments in the history of Egyptian and Babylonian traditional therapies. The historical patterns in permeation analysis are well characterized by Lane and Hadgraft (Hadgraft and Lane, 2005). The skin has developed a crucial route of drug delivery for topical, local, or systemic effects for centuries. Nonetheless, the skin is a significant obstacle and offer hindrances to the transdermal distribution of drugs. Meanwhile, a limited number of drugs can reach the stratum corneum in appropriate concentrations to achieve an active drug quantity in the blood. Numerous methods have been developed and patented to enhance the transdermal absorption of medicines (Barry, 2001; Rizwan et al., 2009). Table 1 highlight the major advantages and disadvantages of TDDS (Escobar-Chávez et al., 2012).

**TABLE 1:** Major advantages and disadvantages of transdermal drug delivery.

Advantages of Transdermal delivery	Disadvantages/Limitation of transdermal delivery
Provide constant plasma levels and is helpful for drugs that need relatively consistent plasma levels (Escobar-Chávez et al., 2012).	The low permeability of the skin limits the number of drugs (Carpentieri-Rodrigues et al., 2007).
Prolong duration of action (Carpentieri-Rodrigues et al., 2007).	Local edema by the drug, Itching, Erythema by the adhesive or other excipients in the patches (Park et al., 2019).
	Irritation at the site of administration.
Decreased dosing frequency (Salinger et al., 2019).	Difficulties in large-scale production.
Provide drug administration flexibility eliminating the patches from the human skin. The alternative route of drug delivery for patients that are not eligible for oral dosage forms.	Disruption of stratum corneum integrity by lipids (Carpentieri-Rodrigues et al., 2007). The possibility of polymorphic transition.

**FIGURE 1:**
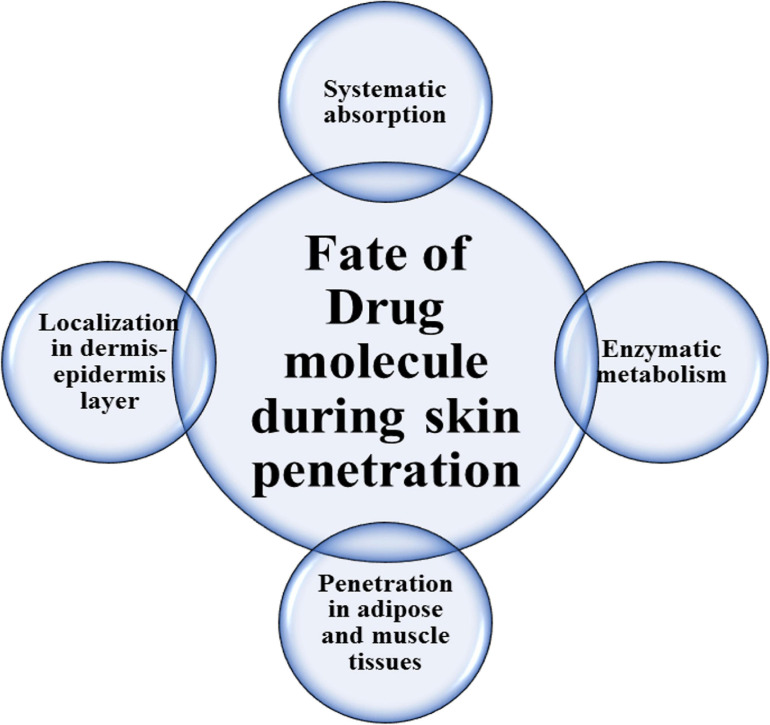
Drug molecules’ fate during skin penetration.

The fate of the medicinal agent in skin penetration through the stratum corneum is shown in Figure 1. Improving physical permeation enhancement technology also contributed to increased interest in the delivery of transdermal medications.

## Skin as a Barrier for Transdermal Drug Delivery

Skin is the body’s most important organ and the body’s largest organ consisting of three main layers (epidermis, dermis, and hypodermis) (Hendriks et al., 2006). It performs a range of essential functions, such as defense (biological, mechanical, chemical, and physical) and hydro and thermo-regulation (Roosterman et al., 2006). Skin appears as a central route for drug delivery by allowing topically administered drugs to travel via stratum corneum (SC) to extra locations inside the skin, which could contribute to the systemic circulation (Sala et al., 2018). Its wide surface area improves drug preoccupation, and thus larger quantity of drugs could be efficiently distributed through transdermal delivery systems. Nevertheless, the SC of the skin also prevents the engrossment of some types of low permeable and/or heavy molecular weight drugs (Schroeder et al., 2007; El Maghraby et al., 2008).

The skin’s barrier function is almost entirely and quite remarkably accomplished by the stratum corneum (SC)- a morphologically and compositionally unique biomembrane (Scheuplein and Blank, 1971). This enormously thin (nearly 1/100 of a mm), least permeable skin layer is the ultimate stage in the epidermal differentiation process, forming a laminate of compressed keratin-filled corneocytes anchored in a lipophilic matrix (Christophers, 1971; Elias, 1981, 1983). The lipids of this extracellular matrix are distinct in many ways (Gray et al., 1982; Williams and Elias, 1987) i.e., (i) they provide the sole diffusion pathway and continuous phase from skin’s surface to the base of SC; (ii) the composition of skin is unique among biomembranes and of particular interest is that it is devoid of phospholipids; (iii) despite this deficit of lipids, the SC lipids produce multilamellar sheets; and (iv) the long-chain, mostly saturated hydrocarbon tails provide an ordered, interdigitated configuration and the formation of gel-phase membrane domains as opposed to the more usual liquid crystalline membrane systems.

**FIGURE 2:**
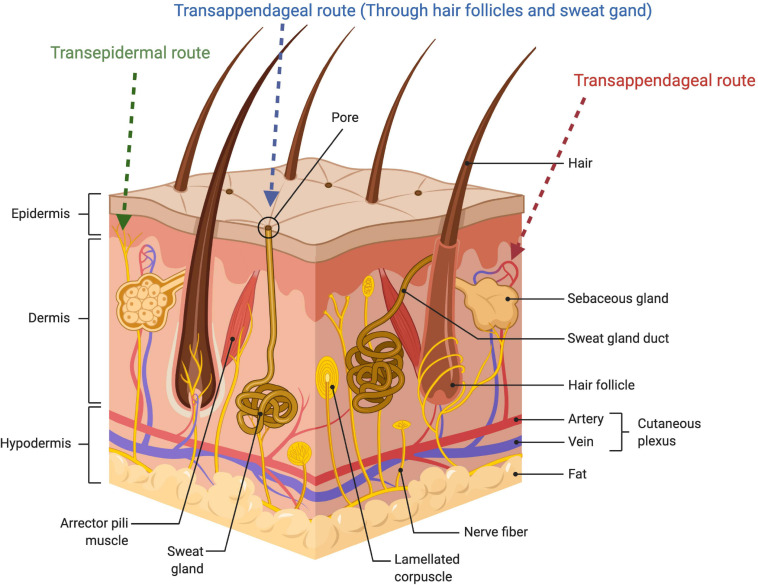
Schematic illustration of the skin layer and showing penetration routes of the drug administered through the skin.

However, it seems that the unusual lipid matrix alone cannot entirely explain the outstanding resistivity of the membrane, and the SC architecture as a whole has been proposed to play a key role in the barrier function of the skin. Hence, the staggered corneocyte arrangement in a lipid continuum is suggested to confer a highly twisted lipoidal diffusion pathway making it 1000-times less permeable to water compared to other biomembranes (Potts and Francoeur, 1991). The transport role of this twisting pathway is further elucidated by visualization studies including localization of different permeants in the intercellular channels (Nemanic and Elias, 1980; Bodde et al., 1991), kinetic analysis of the *in vivo* skin penetration rates of different model compounds (Albery and Hadgraft, 1979), and through evidence from lipid domains’ thermotropic biophysical studies (Golden et al., 1987; Potts and Francoeur, 1990). Figure 2 shows the schematic view of different skin layers.

## Routes of Drug Penetration via Skin

Various studies have been conducted to determine the route of penetration of topical compounds via the skin. The permeability of drugs to the skin essentially require that the retained epidermis should be diffused by the drug. The human skin additionally contains sweat glands and hair follicles, which form thrust pathways via the intact epidermis, accounting for 0.1% of the entire skin (Illel, 1997). The penetration of the drug through the skin is often regulated by the stratum corneum. Two different pathways namely transcellular and intercellular are suggested for drugs to cross this complete barrier.

### The Intercellular Lipid Route

The stratum corneum (SC) possesses interlamellar areas, such as linking regions, ordered lipids, and stable hydrophobic chains providing a non-planar gap between both the neighboring cells and the crystalline lipid lamellae of the cell wall. For transepidermal diffusion of lipid and amphiphilic particles, fluid lipids in the skin layer are of vital significance, filling these spaces for migration and insertion (Geinoz et al., 2004; Ly and Longo, 2004). Hydrophilic molecules diffuse mostly “laterally” through surfaces with less inter-lamellar spaces filled with water or across certain volumes. Polar molecules can also use the available spaces at the same end between the outer membrane and the corneocyte of the lamella (Gupta and Trivedi, 2016).

### The Transcellular Pathway

The intracellular macromolecular matrix runs very deep in keratin, which does not directly add to the diffusive barrier of the skin; however, it promotes mechanical stability and thus contributes to stratum corneum intactness. Transcellular diffusion is unrelated to TDD (Cevc and Vierl, 2010). Confocal microscopy has been applied to track small aqueous transepidermal pathways where regions with intercellular and low molecular weight lipids overlap with skin wrinkles. The surface and, at the same time, the areas with the lowest lipid density resist the delivery of hydrophilic substances including drugs. Among corneocyte clusters, this small resistance pathway occurs in positions where there is no lateral similarity between such groups. The more confined and transport resistant mechanism is the intra-cluster/inter-corneocyte mechanism (Schätzlein and Cevc, 1998). The hydrophilic duct has openings of ≥ 10 nm (narrow pores of intercorneocytes) and ≥ 5 μm (appendages of the skin). Thus, they form the highest width/lowest resistance end of the continuum are pilosebaceous units (5–70 μm) and sweat ducts (≥50 μm), sebaceous glands (5–15 μm). Cluster boundaries and junctions of clusters collapse within the range (Mitragotri, 2003). The lipophilic cutaneous boundary is defined by the predicted values and molecular weight rather than the molecular size of the drug (Johnson et al., 1997). Different tools are applied for measuring the number of drugs found in the skin or different skin layers. It has not been possible to accurately and non-invasively quantify the volume of topically used materials absorbed into the follicle. As a result, cyanoacrylate skin surface, stripping techniques, peel biopsy, etc., have been used to extract the topically applied dye-containing stratum corneum components and estimate the amount of drug absorbed through the transdermal route (Nguyen et al., 2019). Different stripping techniques have also been applied to test the delivery of topically used products selectively and non-invasively into the follicular infundibula (Desai et al., 2013; Nakkam et al., 2018; Carolina Oliveira Dos Santos et al., 2019).

## Drug Properties and Transdermal Penetration

Percutaneous drug absorption depends on physico-chemical characteristics - which have the capacity of facilitating absorption as well as on the condition of the skin. The factors that influence percutaneous absorption include daily dose, molecular weight, lipophilicity, and melting point. Ideally, the daily dose of the drug should be less than or equal to 20 mg per day, molecular weight should be less than 500 Da, should have a log *P*-value between 1–3 and the melting point should be less than 200°C. Moreover, the drugs applied to the transdermal delivery route should be non-irritating and non-immunogenic (Finnin and Morgan, 1999).

In general, biomembranes consist of a lipophilic membrane with a hydrophilic portion. The fundamental equation which described passive drug transport through biomembranes is based on Fick’s first law:

J=P⁢x⁢C⁢a⁢q

where *J* is the flow of the drug through the membrane (mass/area/time), *P* is the permeability coefficient through the lipophilic membrane and *C* is the concentration of the drug between both sides of the membrane. The permeability coefficient is defined as:

P=DxKh

where D is the drug’s diffusion coefficient within the membrane, K is the partition coefficient of the drug from the external environment towards the membrane and h is the thickness of the membrane. Finally, the diffusion coefficient can be estimated by the Stokes-Einstein equation:

P=RT6πηrN

where R is the molar gas constant, T is the absolute temperature, η is the viscosity of the membrane, r is the radius of a spherical drug molecule permeating the membrane, and N is Avogadro’s number. The equation demonstrates that in order for the drug to be released across the membrane, it must present some aqueous solubility, (C_*aq*_); however, at the same time, it must also possess lipophilicity and suffer partition from the external aqueous environment to the lipophylic membrane (K). By observing Stokes-Einstein equation, it’s possible to see that small molecules are able to permeate much more easily than large molecules (Loftsson, 2002).

The hydration of the skin is another factor that influences the percutaneous absorption of drugs. The greater the hydration, the higher and better the absorption of substances, in particular polar substances. Hydration is the result of the diffusion of water from adjacent layers, or of an accumulation of water – after the application of some material and/or occlusive vehicle. Other factors, such as the area and method, region, period of application, the age of the skin, and the use of vehicles which alter barrier function also influence percutaneous absorption (Idson, 1975).

In a parallel manner, the presence of determined compounds, which act with diverse mechanisms, can provide variations in flow, due to modifications in cellular membrane structures, diffusion coefficient alterations, the partition coefficient of the SC/vehicle, influences cutaneous water content, and can also modify superficial tension. These substances are designated modifiers, accelerators, catalyzers, or simply, absorption promotors. Such substances or physical methods, usually temporarily and reversibly modify the SC barrier, assisting in drug penetration. Iontophoresis, electroporation, and ultrasound - physical methods -, apart from diverse chemical absorption enhancers are used to make this route viable as a drug administration alternative (Carpentieri-Rodrigues et al., 2007).

## Overcoming the Functional Barrier of Skin for Transdermal Drug Delivery

The skin exhibits a surprising evolutionary effect which not only includes the physical penetration of microorganisms and multifunctional interface with surroundings but also involves the assembly of a highly effective hemostatic barrier to prevent water loss. To do so, the skin serves as an adept membrane to limit the transportation from the body to the environment or vice versa. This barrier is considered as the main challenge for the scientists working in the field of transdermal drug delivery. To overcome the skin barrier, an efficient approach is highly required to enhance the drug permeability through the skin and to expand the drugs’ range for transdermal application. To achieve this goal, various strategies based on varying complexity and efficacy are available for the pharmaceutical scientists which are described below (Naik et al., 2000):

### Passive Penetration Enhancement

The simplest approach involves the manipulation of the formulation used in the transdermal drug delivery. While the design of super-loaded formulation, i.e., supersaturation, microemulsion, and liposomal systems, is the more innovative approach to overcome the skin barrier (Osborne et al., 1990; Davis and Hadgraft, 1993). These approaches have modestly improved the penetration across the membrane but are unlikely to transform an impermeable drug entity into an ideal transdermal candidate.

#### Drug Modification

Considering the drug molecules, the process of derivatization can be used to modify the lipophilicity of any drug candidate. This approach is involved in the pro-drug strategy to physically modify the drug candidate for enhanced skin permeation. This prodrug is enzymatically converted into its active form (Lee and Li, 1989). Hence the prodrug approach is successfully being utilized in the field of the topical steroidal anti-inflammatory agent where the inherent property of the pharmaceutical molecule is not often exact with the topical potency. For instance, triamcinolone has five times greater activity than that of hydrocortisone but exhibits very low topical activity i.e., only 10%. However, the topical efficacy of triamcinolone increase by 1000-folds after being transformed into acetonide as this modification provides favorable lipophilicity to triamcinolone. Similarly, the topical activity of betamethasone was increased by 450-folds after conversion into 17-valerate analog via esterification. This approach has widely been exploited to develop a transdermal formulation and the pharmaceutical scientist are in continuous efforts on the method development to improve the permeability of the membrane *per se* (Naik et al., 2000).

#### Chemical Enhancement

One of the widely utilized approaches includes the use of chemicals which can reversibly alter the function of the skin’s barrier and therefore allow penetration of low permeable drug candidates into the membrane and systemic circulation. Various chemicals have been reported to make the SC more permeable including poly-alcohols, alcohols, amines, pyrrolidones, amides, sulphoxides, fatty acids, alkanes, esters, surfactants, terpenes, and phospholipids, and much more (Walters and Hadgraft, 1993). The structural diversity of these molecules prevents the formulation of any singular mechanistic hypothesis but it remains a source of debate which one is well echoed by the vast penetration-enhancer through literature. However, mechanistic information has been reported for some of the molecules at the molecular level with the variable mode of action, i.e., alcohols (e.g., ethanol) enhance the permeation by extracting and solubilizing the lipid content of SC (Kai et al., 1990), while fatty acids (e.g., oleic acid) are involved to induce phase separation and lipid fluidization within the membrane (Ongpipattanakul et al., 1991; Naik et al., 1995). On the other hand, simple hydration is a well-known method to enhance the absorption without affecting the conformation of lipid bilayer or spacing but the mechanism is not yet known (Lieckfeldt et al., 1994). Penetration enhancer 1-dodecylazacycloheptan-2- one (Azone), show its activity at lower concentration and the possible mechanism for its performance involves lipid fluidization and ion-pairing (Hadgraft et al., 1985). It has also been reported that the enhancers (e.g., fatty acids, Azone) and more polar co-solvents (e.g., propylene glycol, ethanol) synergistically affect the solubilization of the constituents within the SC hence promoting the lipid-modulating effect. Likewise, the absorption mechanism of solvents such as Transcutol involves the solubilization of drugs within the membrane rather than causing the increase in drug diffusivity (Harrison et al., 1996). By contrast, sulphoxides, are comparatively aggressive and capable to induce various structural deterioration, i.e., solubilization of membrane components and keratin denaturation thus act as permeation enhancers.

### Physical Penetration Enhancement

Transdermal systems provide several advantages, one of them is the control and sustain release of drug candidate suffering from the short biological half-life and require frequent administration. The newly developing ‘biotechnology’ drugs (i.e., proteins, peptides, and oligonucleotides) are particularly prone to this problem. Although these therapeutics are highly specific and potent but cannot be delivered by conventional methods because of their exceptionally labile nature (Khavari, 1998; Pettit and Gombotz, 1998). Besides this, these drugs are usually large molecules with polar and/or charged groups which significantly limit their transdermal delivery. However, physical enhancement technologies (described below) are working to directly respond to the described challenges and offer dominant and exciting strategies to overcome the issues of transdermal application (Berner and Dinh, 1998).

#### Iontophoresis

Iontophoresis is the most evolved technology facilitating drug transportation across the membrane by using a small electrical current (usually, 500 microamperes cm^2^). The electrical potential allows the transport of charged species into the skin but the efficacy of this approach is strictly dependent on the ionic mobility, polarity, and valency of the drug being permeated as well as on the constituents of the drug delivery formulation. Typically, two electrolyte chambers (cathodic and anodic) are employed on the skin and operate by using a constant current source. The anodal chamber is contained the cationic drug while the other contains the ionized molecule of the therapeutic drug having the same polarity. The transportation of ions into the membrane is dependent on the magnitude of current because it is associated with the charge generation within the circuit which in turn transports the charged ions into the skin. This approach is considered an efficient and controlled drug delivery method because the delivered drug content is directly related to the applied charge intensity (Guy, 1992).

At the physiological pH, the skin carries a net negative charge hence the delivery of the cationic drug is generally preferred because the negatively charged membrane selectively accepts the cationic substances under the influence of an applied electrical field. However, transportation of small cations (such as buffer salts) also occurs in the direction of the movement of cations (anode to cathode). During their path, these ions collide with solvent molecules and result in an electro-osmotic flow which subsequently permits the transport of neutral species (along with cationic substances) that can now literally be transported from the anode to the cathode. For the transportation of cationic drugs, with increasing molecular weight, the contribution of electro-osmosis becomes more significant as compared to the classic electro-repulsive effect such that it is probably considered as the main transport mechanism for peptides and small proteins via iontophoresis (Guy et al., 2000).

Over passive transport, iontophoretic transdermal delivery exhibits various advantages such as dosing precision potential, and pulsatile delivery profiles, which allow endogenous release patterns to be mimicked and the avoidance of unwanted effects (e.g., tolerance) as a result of sustained drug input. Iontophoresis presents an authentic protocol for the administration of polar and moderately sized (up to 7 kDa) ionized molecules (Guy, 1998). The delivery of such molecules at therapeutic levels is a function of both the transportability and potency of the molecule [e.g., although iontophoretic delivery of insulin (6 kDa) is possible the obtained plasma concentrations are not enough which can fulfill the requirement of diabetic patients. Hence, intense research is required for the transcutaneous route followed by the molecules delivered by iontophoresis. Certainly, up-to-date research showed the higher significance for this method of drug delivery besides that some other pathway might be created after the transportation of molecules under the high electric influence as the charged permeants try to follow the path of least resistance (Scott et al., 1992; Jadoul et al., 1995).

#### Electroporation

Electroporation uses high-voltage pulses for a shorter duration of time and it is believed that this process creates the permeabilizing potential at the localized regions by making the aqueous pathway in the bilayer lipid membrane (Tsong, 1989). Based on this destabilizing tool, permeabilizing nuclear membranes to effect DNA transfer currently has become a topic of interest to enhance transdermal transport (Prausnitz et al., 1993). *In vitro* analysis has been performed for the electroporation of the SC using exponential voltage pulses or square waves that are capable to produce a transmembrane potential of up to 1 kV with a time of about 10 ms–500 ms. This approach of electrical transdermal improvement is considered as most effective (quantitatively) than that of iontophoresis as confirmed by the *in vitro* analysis (Bommannan et al., 1994). Furthermore, electroporation significantly enhances the transport level as compared to passive delivery (Wang et al., 1998), but it is limited *in vivo*, and skin toxicological studies are present hence the establishment of clinical value is still required.

#### Ultrasound

Ultrasound, the sound of frequency greater than 20 kHz, is generally used to alter the skin’s barrier function (Barry, 2001; Escobar-Chávez, 2016). Although ultrasound technology (sonophoresis) is widely used in various diagnostic procedures and physical therapy, its unambiguous consignment to the ‘enhancement armamentarium’ is yet to be understood. The frequencies that have been reported to enhance the transdermal delivery cover a range of 20 kHz to 10 MHz with intensities of up to 3 W cm^2^. It is reported that as compared to conventional means, low-frequency ultrasound (∼20 kHz) is capable of the greater deterioration of the skin barrier as the therapeutic ultrasound (∼1 MHz) treatment caused the 1000-fold enhancement in transportation (Mitragotri et al., 1995b).

Mechanistically, sonophoresis creates various amendments in the skin tissues (thermal, chemical, and mechanical alterations) to enhance drug delivery. Sonophoresis is also reported to form small gaseous pockets within cells (cavitation) along with the elevation of skin temperature. The increase in pore size causes the increased alterations of SC lipid architecture (Mitragotri et al., 1996), which may eventually lead to significant cytotoxicity (Bommannan et al., 1992). However, cavitation is considered as the main mechanism through which low-frequency ultrasound facilitates the skin permeation and probably accounts for the improved transportation of polar macromolecules such as interferon-g (∼17 kDa), insulin (∼6 kDa), and erythropoietin (∼48 kDa) through the *in vitro* model of human skin (Tachibana, 1992). Insulin has been successfully delivered to the skin of the rats and rabbits (*in vivo)* via ultrasound-mediated transdermal delivery (Tachibana, 1992; Mitragotri et al., 1995a). The study provoked curiosity but data still needs validation on human models to achieve a safe and effective level of ultrasound application.

## Nanocarriers for Transdermal Drug Delivery

**FIGURE 3:**
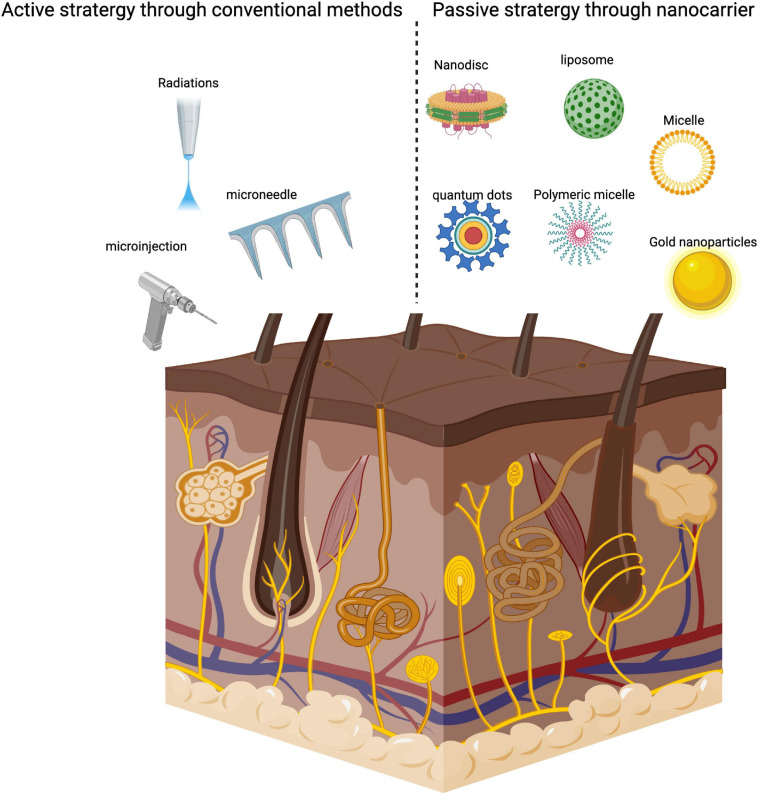
Drug delivery strategy of Nanocarriers and conventional approaches.

Nanocarriers are classified as colloidal structures with a mean diameter of fewer than 500 nanometres (Neubert, 2011). New nanocarriers, including liposomes, nanoparticles (NPs), nanoemulsion, and microemulsion are most investigated for TDD. NPs have several benefits, such as higher drug diffusion for TDD systems in the target area, improved physicochemical stabilization of the drug-loaded in nanoparticles, and sustained and regulated drug delivery. Lipid-based NPs including liposomes, solid lipid nanoparticles (SLNs), niosomes nanostructured lipid carriers (NLCs), and nanoemulsions have also been widely used for TDD delivery (Patzelt et al., 2017). Hair follicles act as a significant route to improve skin penetration for both transdermal and dermal delivery. The mean size of NPs is by far the most significant factor that decides the extent of follicular penetration independent of the form of NPs (Patzelt et al., 2017; Ghasemiyeh et al., 2019). Hair follicles targeting might be an awesome option for the treatment of hirsutism androgenetic alopecia, and acne vulgaris. Transfollicular drug delivery does have the benefits of prolong drug accumulation and storage, deep skin penetration, targeting the tissue, and improved skin bioavailability (Fang et al., 2014). However, transdermal hair follicle delivery is correlated with several challenges such as low physical properties of activity, including compatibility, pharmacokinetics, metabolism, and solubility, Paudel et al. (2010), which need to be tackled. Compared to the traditional drug delivery approaches, nanocarriers offer a passive drug delivery strategy (Figure 3) which is considered to be safer and quicker than the conventional methods.

### Solid Lipid Nanoparticles (SLNs)

Solid lipid nanoparticles are produced by emulsifiers and solid lipids. These are made from lipids that are fully stable at room temperature (Garcês et al., 2018a). SLNs are nanocarriers with sizes ranging from 50 to 2000 nm. Lipid NPs are recognized as secure drug delivery systems for a variety of reasons, including biodegradability, low toxicity, and biocompatibility (Ghasemiyeh et al., 2017). SLNs based TDD systems have the potential for occlusive and adhesion characteristics that allow them to create a homogeneous and even coating on the stratum corneum and extend the amount of residence and boost the absorption through the skin (Arora et al., 2017). TDD through lipid NPs could result in high drug deposition in particular target areas such as hair follicles, sweat glands, and sebaceous glands and reduce systemic adverse reactions (Borgia et al., 2005). Studies also reported that SLNs could dramatically improve the dermal absorption of active pharmaceutical products relative to simple o/w creams and nanoemulsions owing to SLN occlusion. *In vivo* research has shown that cyclosporine A and calcipotriol-loaded lipid NPs had the highest decrease in inflammation of the eye, increased lesion, and lower psoriatic score relative to free medicines (Arora et al., 2017). Loteprednol etabonate (LE) is a carbon-20 ester corticosteroid having potent anti-inflammatory effects and a reduced propensity for provoking corticosteroid related adverse effects. It has been demonstrated that reducing the drug particle size to the nanometer range in diameter using SLNs provides effective ocular tissue penetration and resolution of pain and inflammation despite a reduced drug concentration (0.38%) and dosing frequency (Salinger et al., 2019). Benzoyl peroxide SLN has demonstrated higher drug accumulation in lower skin inflammation, skin organelles, continuous release of drugs, lower systemic absorption of drugs, and decreased adverse effects relative to the current marketing formulations (Pokharkar et al., 2014). Lipid NPs are significant carriers for lipophilic drugs for topical delivery having high log *P*-values (log*P* > 3) and maximum molecular weight (≤500). The term “cutaneous absorption” is properly used to characterize the sum of the amounts of a drug that penetrates and permeates the skin. The amount of drug absorbed through the skin depends on many factors including the nature of the drug, the drug load in the nanocarriers, type of surfactants or other permeation enhancers used, and the area on which the formulation is applied. Thus different formulations have respective capabilities of delivering the loaded drug in different amounts (Ruela et al., 2016). Owing to their lipid nature, SLNs are more effective at trapping and penetrating deep into the skin layers. The selection of surfactant mixtures and lipid plays an essential part in lipid nanoparticle drug encapsulation, especially SLNs (exhibits suitable crystalline structure) in the prevention of drug leakage throughout processing and delivery. The absorption of drugs in the lipid mixture should also be measured before the manufacture of nanoparticles (Arora et al., 2017).

A most critical drawback of topical SLNs is their ideal crystalline form, which leads to higher bursting effects and low drug loading. To tackle this restriction, the application of a second lipid portion to the solid lipid matrix has recently demonstrated improved stability, increased drug loading performance, and improved prolong release potential (Chantaburanan et al., 2017). Lipid-based NPs systems may have a thick film on the skin surface with an occlusive effect and improve the hydration of the skin. The involvement of surfactants in this system can loosen or fluidize the stratum corneum and can enhance the permeation of loaded drugs (Fang et al., 2008). SLNs can also be employed in cosmetics, e.g., the anti-wrinkle impact of retinyl palmitate-loaded SLNs has been tested and the findings showed that it can protect the skin and improve skin penetration.

### Nanostructured Lipid Carriers (NLCs)

Nanostructured lipid carriers are produced by the combination of liquid and solid lipids (Garcês et al., 2018b). Common liquid lipids used for the preparation of NLCs are Triolein (Ghasemiyeh et al., 2017), Miglyol (Stecová et al., 2007), oleic acid (Stecová et al., 2007), sesame oil, Copaiba oil, Capryol PGMC, and sweet almond oil (Ribeiro et al., 2017), to mention a few. These nanoparticles are actual carriers for medicinal and cosmeceutical products for dermal, transdermal, and follicular applications. The usage of these nanocarriers have numerous benefits, for example, drug safety, controlled release of drugs, enhanced bioavailability of drugs, and improved diffusion and deposition in the tissues. The potential mechanism of NLC to attract skin penetration is its greater occlusion, which can be achieved by inducing an improvement in the angularity of nanoparticles and stratum corneum within a precise surface area, as well as the volume and degree of encapsulation of loaded molecules.

Lipid nanoparticles have the possibility of interfering with the lipid bilayer and persuading lipid reorganization that can increase the accumulated drug molecules’ penetration. NLCs also recover skin hydration by stopping trans-epidermal water loss and through film starting on the stratum corneum surface (Schwarz et al., 2013). Other promising applications of lipid nanoparticles (especially NLC) are in the delivery of active drugs through follicles to address androgenic skin diseases, such as acne, hirsutism, and hair loss. The important property of nanocarrier impacting the volume and depth of follicular distribution is the size of nanoparticles (Ghasemiyeh et al., 2017), i.e., tiny elements can enter the blood, while larger particles stay on the stratum corneum surface and intermediate nanoparticles can be deeply focused in sebaceous glands and hair follicles (Ricci et al., 2005). In cosmetics, NLCs showed good properties than SLNs, such as octyl methoxycinnamate (OMC)-loaded NLCs demonstrated better UV safety than WTO-loaded SLNs, which may be credited to maximum WTO solubility in liquid NLC lipids (Garcês et al., 2018a).

### Liposomes

Liposomes are made of unilamellar or multilamellar lipid bilayers surrounding an aqueous phase. Liposomes are usually composed of phospholipids and cholesterol. Liposomes can act as vesicular nanocarriers and have many benefits for TDD, including local anesthetics in the epidermis, controlling drug release, enhancing absorption, and reducing side effects. It has been shown that local tissue activation of liposomes reduces blood pressure and urination (Maghraby et al., 2006). The deposition performance of liposomes’ encapsulated drug is strongly influenced by the composition of the liposome, particle size, lamellarity, fluidity, and occlusive characterization, and also relay on liposome preparation methods. SCL (stratum corneum liposomes) have been documented to have improved sedimentation stability compared to the alcohol-oil emulsions, and hydroalcoholic solutions have greater precipitation compared to phospholipids (Egbaria et al., 1990; Lee D. W. et al., 2015). Another application of liposomes as a local drug delivery mechanism is their possible follicular targeting (Lieb et al., 1992). Liposomes have the potential to produce TDD, and current studies have shown that deformable vesicles (called transdermal vesicles) are a safer alternative to traditional liposomes for transdermal delivery (Maghraby et al., 2006). Adding ethanol to the composition of liposomes (so-called ethosomes) improves the durability of the vesicles, and therefore the efficiency for skin penetration.

Super-deformed sulforaphane-loaded vesicles have been extensively investigated for treating certain cancers. Its advantage is that it reduces the dose compared to the traditional delivery routes, thus minimum adverse effects are observed. Elastic liposomes having a particle size of less than 300 nm have also been investigated for the treatment of skin inflammatory diseases and skin cancer (Di Francesco et al., 2017). These types of nanocarriers have the advantages of prolonging stability and improved drug release kinetics. Though there are many pitfalls in the delivery of liposomal drugs via skin due to stability and penetration problems (Cristiano et al., 2020). Paclitaxel-loaded liposomes have been tested as a possible treatment for squamous cell carcinoma in a study. Their findings indicate that compared with free drugs, this TDD device can increase skin penetration via the stratum corneum and enhance its anti-proliferative effect, which may help improve the treatment of cases of squamous cell carcinoma (Paolino et al., 2012). Liposomes have two main functions in TDD e.g., deposition and preservation (directed delivery to skin organelles), and enhancement of drug penetration (Choi and Maibach, 2005). The particle size of liposomes is an important parameter for skin penetration, as previous studies have shown that smaller liposomes can retain water in the skin layer, and smaller unilamellar vesicles (SUVs) may be more effective than multicellular vesicles. Liposomes larger than 600 nm cannot be effectively transmitted to the skin layer, and most of them adhere to the surface of the horn layer. Liposomes with a particle size of 300 nm penetrate deeper into the skin layer, while liposomes of 70 nm are promising for skin delivery (Choi and Maibach, 2005).

### Niosomes

Niosomes are single or multi-layer spheroidal structures made of surfactants and considered the counterpart of liposomes (Balakrishnan et al., 2009). Nonionic surfactants are safer, fairly non-toxic and biocompatible with the host, and can act as a thickening agent in TDD. Surfactants that exhibit increased HLB values are not capable to form a vesicle due to the advanced hydrophilicity of their particles, however, Tweens and Spans are ideal non-ionic surfactants for the preparation of noisome (Tavano et al., 2017). One of the processes for enhanced permeation of niosomes through the skin is the elimination of trans-epidermal water loss (TEWL) while the alternate mechanism is the adsorption or fusion of the vesicles with skin lipids.

Niosomes can disturb the configuration of the stratum corneum to render it looser and more permeable (Muzzalupo et al., 2017). Non-ionic surfactants used in niosomes formulations can even serve as ideal enhancers of skin permeation (Choi and Maibach, 2005). The tiny size of the niosomes as drug-delivery devices can even improve the bioavailability of the encapsulated medicines through the skin. Previous research has shown that minoxidil-loaded niosomes could dramatically improve skin penetration and skin bioavailability relative to the conventional topical formulation. It has also been reported that particle size and surfactant behavior had a substantial effect on the amount of skin permeation and bioavailability of encapsulated medicines. Previous research indicated that 5-aminolevulinic acid (ALA)-loaded niosomes could dramatically increase the penetration of the drug relative to basic aqueous suspension (Bragagni et al., 2015). Niosomes can function as nanocarriers for chemical drugs and peptides and proteins. Spans and Tweens are the most commonly used surfactants for niosomes for drugs, while diacyl glycerides and poly-oxyethylene ether are mainly used in the niosomes for the delivery of peptides and proteins. These factors can also serve as permeation enhancers in the formulations of niosomes (Choi and Maibach, 2005). The types of non-ionic surfactants and the amount of cholesterol in niosomes are important parameters for transdermal delivery. Previous studies have suggested that Tweens can improve transdermal delivery more than Span and lower levels of cholesterol can improve transdermal delivery through niosomes (Fang et al., 2001). Vesicular membrane fluidity is an essential factor influencing the delivery of transdermal drugs by niosomes. Essential oils, in particular terpenes, are considered an essential element of niosomes formulations that can serve as penetration enhancers by disrupting the structure of the stratum corneum. Besides, essential oils have the ability for vesicular fluidization and improvement of the elasticity of the niosomes, which may increase the delivery of transdermal drugs. A previous study has demonstrated that the addition of essential oils to the formulation of felodipine-loaded niosomes greatly improved the permeation of the drug and this improvement was heavily influenced by the quantities and types of added essential oils. Lemon oil, clove oil, and eucalyptus oil may dramatically improve transdermal distribution relative to free niosomes (Eid et al., 2019).

### Nanocrystals

Nanocrystals are particulate structures that are entirely made up of drug particles through top-down or bottom-up approaches. Nanocrystals vary in size from 1 to 1000 nm (Pireddu et al., 2016) and are kept dispersed and stabilized by stabilizers to prevent their aggregation. There are three kinds of stabilizers which are commonly used for nanocrystal development, including ionic stabilizers, non-ionic stabilizer; and polymeric stabilizers (Müller et al., 2011). The topical formulations of drug nanocrystals can allow increased dermal bioavailability of the drug, saturation solubility, surface adhesion, drug release rate, and dissolution rate (Shegokar, 2016). Milling approaches and high-pressure homogenization techniques (top-down) are the most common approaches used in nanosuspension and nanocrystal formulation. The final aim of nanotechnology is to increase the absorption of poorly water-soluble drugs in the skin. A previous study has reported that lutein nanocrystals (nanosuspension) display remarkably greater saturation solubility compared to the lutein microcrystals (coarse powder and the nanocrystals had improved skin penetration (Vickers, 2017).

As nanocrystals can improve the dissolution rate of poorly water-soluble products, they are an efficient new delivery system for dermal use. Nanocrystals are effective drug carriers for water-insoluble drugs and drugs with minimal skin penetration, however, the most significant drawback is their optimization for size and regular dosing (Döge et al., 2016). Nanocrystals are used to boost skin deposition, increase skin permeation and achieve rapid skin penetration compared to standard topical formulations. Research has shown that dexamethasone nanocrystals significantly improved skin penetration compared to traditional dexamethasone creams and dexamethasone-loaded ethyl cellulose nanocarriers. These findings also showed that much of the medication was deposited in the dermis layer in nanocrystal formulations relative to traditional nanocarriers in which much of the drug stayed in the epidermis and rapid skin permeation was observed for nanocrystal (Pyo et al., 2017).

### Natural Lipids Based Nanoparticles

Lipid nanoparticles may be formulated using natural lipids like Illip butter with the potential for skin hydration and Calendula oil with the benefit of anti-inflammatory and curing properties. These natural lipid nanoparticles can be used to encapsulate certain active pharmaceuticals for selective skin delivery (Pivetta et al., 2018). Another benefit of natural lipid nanoparticles is their lower potential toxicity relative to other synthetic ones. An example of these natural lipids is stearin fractions of fruit kernel that have been examined in the topical drug distribution of tretinoin (Mandawgade and Patravale, 2008). Natural nanoparticles will also be regarded as potential nanocarriers for skin distribution and targeting strategies of high effectiveness and reduced toxicity issues. Table 2 enlists the various therapeutic agents loaded in nanocarriers along with their benefits and drawbacks.

**TABLE 2:** Therapeutic materials encapsulated in nanocarriers and their applications.

Nanocarriers (size)	Advantages/Disadvantages	Active Drug	Applications	References
Niosomes (100-2000 nm)	Can be used for both hydrophilic and lipophilic drugs, relatively stable/stability issue, irritation	Quercetin	Antioxidant and skin whitening agent	Lu et al., 2019
		Asiaticoside	Antipsoriasis, antiaging, and burn and wound healing agent	Wichayapreechar et al., 2020
Liposomes (20-10,000 nm)	Can carry both hydrophilic and lipophilic drugs/shelf life, stability issue, costly	Vitamin D3	UV protective and antiaging agent	Bi et al., 2019
		Folic acid	Skin regenerative and nutritional Agent	Kapoor et al., 2018
Nanostructured lipid carriers (10-1000 nm)	enhanced drug encapsulation and stability/often Lipid particle growth	Hydroquinone	Antioxidant and skin bleaching Agent	Wu et al., 2017
		Isoliquiritigenin	Antioxidant and skin whitening agent	Noh et al., 2017
Nanoemulsions (50-200 nm)	Can be used for all type of lipophilic drugs/irritation, stability issues, phase separation, and inversion	docosahexaenoic acid and Eicosapentaenoic acid	Anti-proliferative and Anti-inflammatory	Kabri et al., 2011
		Hyaluronan	Hydrating and antiaging agent	Kong et al., 2011
Solid lipid Nanoparticles (50-100 nm)	Stable, prolong the release of drugs/unpredictable gelation, low drug encapsulation	Tazarotene	Acne scare healing, Antipsoriasis, and photo-aging properties	Rajkumar et al., 2019
		Idebenone	Antioxidant and hydrating agent	Montenegro et al., 2018

## Combination of Nanocarriers and Physical Methods for Enhanced Transdermal Delivery

Ultrasonic treatment (sonophoresis, phonophoresis) is one of the less invasive physical enhancers (Tyle and Agrawala, 1989; Mitragotri, 2017). It is effectively utilized for enhanced intra- and transdermal delivery of bioactive molecules (Mitragotri, 2017) and particulate systems (Genina et al., 2019). Such ultrasonic treatment of skin at frequencies higher than 0.7 MHz gives rise to pressure changes in the medium, forming cavitation bubbles inside the inherent cavities represented by hair follicle shafts and sweat glands (Polat et al., 2011). Bubbles oscillation in the follicles push the particle suspension down the follicle. It was shown, that application of 1 MHz ultrasound at a power density of 2 W/cm^2^ did not induce adverse effects on rat skin (Levy et al., 1989). Prolongation of the enhanced permeability time window allows for the stepwise application of drugs or vaccines (Mitragotri et al., 1996). Ultrasonication can also be coupled with other methods. Thus, enhanced transdermal delivery of solid nanoparticles by simultaneous application of ultrasound and various chemical enhancers has been demonstrated (Lopez et al., 2011; Zaytsev et al., 2020).

Iontophoretic delivery is another effective non-invasive approach for therapeutic drug delivery (Merino et al., 2017; Wu et al., 2020). Generally, transdermal iontophoresis creates a small electric current (0.1–0.5 mA/cm^2^) in the skin to induce transdermal molecular transport, enhancing drug transportation by electrorepulsion and electro-osmosis. At neutral pH, the skin is negatively charged and cation-permselective (Burnette and Ongpipattanakul, 1987). Thus, the current passage causes a convective solvent flow from anode to cathode, facilitating cation transport and enabling the enhanced transdermal transport of neutral, polar solutes. Relative electrorepulsion and electro-osmosis effects depend on the physico-chemical and electrical characteristics of the membrane and permanent. Besides, the negative charge of the skin can be reduced, neutralized, or even reversed by the iontophoresis of certain cationic, lipophilic species (Hoogstraate et al., 1994).

Simultaneous application of sonophoresis and iontophoresis has also been applied (Long et al., 2000; Watanabe et al., 2009; Park et al., 2019). The synergistic effect of their application is demonstrated for transdermal delivery of various cosmeceutical drugs using a Franz diffusion cell. Such a combined strategy is advantageous as it declines the energy density and thereby reduces skin irritation (Park et al., 2019).

Phono- and iontophoresis were successfully applied for translingual delivery of antimycotics (e.g., terbinafine and ciclopirox) allowing the significant enhancement of their penetration into nail plates (Nair et al., 2009; Kline-Schoder et al., 2019). Heating is another physical approach allowing one to improve the delivery profile of topical medicaments (Hao et al., 2016; Szunerits and Boukherroub, 2018). The heat-enhanced effect is generally attributed to both an increase in drug diffusion in the vehicle and skin and an increase in skin lipid fluidity. Furthermore, the skin temperature rise increases its blood supply that also plays an important role in enhancing the transdermal delivery of a topically applied compound (Szunerits and Boukherroub, 2018).

Microporation is one of the most common invasive physical methods applied for skin barrier removal. Such a method allows the formation of micropores or even microchannels in the skin by the usage of microneedles fabricated of different materials and geometries (Migdadi and Donnelly, 2019; Waghule et al., 2019) or laser and radiofrequency ablations, which allows for further transferring of water-soluble molecules and macromolecules (Sintov et al., 2003; Szunerits and Boukherroub, 2018), as well as particulate systems (Genina et al., 2013; Belikov et al., 2015; Lee W. R. et al., 2015; Genina et al., 2016; Engelke et al., 2018). Such techniques enable the delivery of much larger molecules with much greater fluxes into the skin than other methods and therefore are extensively developed for delivery of insulin (Fang et al., 2004), hormones (Song et al., 2018), vaccines (Weiss et al., 2012), etc.

Although the formation of micron-scale holes within the stratum corneum is a prospective and successfully applied approach towards transdermal drug transportation, its adaptation for antifungal treatment of skin appears unpromising as this procedure is invasive. Furthermore, it should be mentioned here that micro-needling treatment results in the expression of various genes related to epidermal differentiation, inflammation, and dermal remodeling (Schmitt et al., 2018). Thus, microneedles possess their pharmacological activity which may interact with the antimycotic compound.

The combined application of the most efficient physical methods with various nanocarriers has also been investigated demonstrating its superiority in drug penetration enhancement compared to their single-use (Dragicevic and Maibach, 2018). Thus, for example, a particulate drug delivery system based on the use of porous biodegradable carriers appeared beneficial when applied topically together with sonophoresis (Svenskaya et al., 2019, 2020). Meanwhile, no evidence of systemic toxicity enhancement was indicated. The proposed system provided the transportation of immobilized drugs along with the entire depth of hair follicles, its intrafollicular accumulation, and prolonged storage. Such an approach was successfully applied for transdermal delivery of griseofulvin antifungal drugs as well (Lengert et al., 2019). Some examples (Ding et al., 2011; Tomoda et al., 2011, 2012; Taveira et al., 2014; Charoenputtakun et al., 2015; Huber et al., 2015; Qiu et al., 2016; Takeuchi et al., 2017; Teong et al., 2017) of the combined nanocarriers and physical methods are listed in Table 3.

**TABLE 3:** Examples of Nanocarriers combined with physical methods for transdermal drug delivery.

Nanocarriers (size)	Advantages/Disadvantages	Active Drug	Applications	References
Niosomes (100-2000 nm)	Can be used for both hydrophilic and lipophilic drugs, relatively stable/stability issue, irritation	Quercetin	Antioxidant and skin whitening agent	Lu et al., 2019
		Asiaticoside	Antipsoriasis, antiaging, and burn and wound healing agent	Wichayapreechar et al., 2020
Liposomes (20-10,000 nm)	Can carry both hydrophilic and lipophilic drugs/shelf life, stability issue, costly	Vitamin D3	UV protective and antiaging agent	Bi et al., 2019
		Folic acid	Skin regenerative and nutritional Agent	Kapoor et al., 2018
Nanostructured lipid carriers (10-1000 nm)	enhanced drug encapsulation and stability/often Lipid particle growth	Hydroquinone	Antioxidant and skin bleaching Agent	Wu et al., 2017
		Isoliquiritigenin	Antioxidant and skin whitening agent	Noh et al., 2017
Nanoemulsions (50-200 nm)	Can be used for all type of lipophilic drugs/irritation, stability issues, phase separation, and inversion	docosahexaenoic acid and Eicosapentaenoic acid	Anti-proliferative and Anti-inflammatory	Kabri et al., 2011
		Hyaluronan	Hydrating and antiaging agent	Kong et al., 2011
Solid lipid Nanoparticles (50-100 nm)	Stable, prolong the release of drugs/unpredictable gelation, low drug encapsulation	Tazarotene	Acne scare healing, Antipsoriasis, and photo-aging properties	Rajkumar et al., 2019
		Idebenone	Antioxidant and hydrating agent	Montenegro et al., 2018

## Limitations and Future Outlook of Nanocarriers for Transdermal Drug Delivery

Among various challenges for nanocarriers, there is a lack of essential studies and standards, which are required to develop to ensure an appropriate classification, analytical evaluation, toxicological and pharmacological evaluation of these systems. These are critical for the evaluation of the effectiveness of nanotechnology for therapy and diagnostics due to their tiny scale, maximum surface energy, structure, and design. The key benefits of nanocarrier based drugs are that their multifunctional attributes and it is possible to manipulate the functional groups present in the nanocarriers for desired outcomes (Esfand and Tomalia, 2001; D’Emanuele and Attwood, 2005). Owing to their shape and size (1–10 nm), these particles may hold imaging agents, drugs, and may interfere with lipids in membranes to improve permeation as documented for intestinal absorption and cell cultures. Nanocarriers improve the absorption of lipophilic drugs more effectively compared to hydrophilic drugs.

Compromised biodegradation and intrinsic cytotoxicity are the major hurdles with these formulations for translation to clinics (Parekh, 2007). Different strategies have been developed to overcome this issue such as pairing with other biocompatible ingredients. Dendrimers have been paired with peptides to produce less toxic dendrimers. Dendrimers-peptides are composed of amino acids, which are bound by peptide-amide to the branches of dendrimers in the core or upon the upper surface to mitigate toxicity. Moreover, the production of these complexes is less costly and there is little problem in purification (Cloninger, 2002; Niederhafner et al., 2005). Further research is required to study the interactions of nanocarriers as well as other particles, and the interactions of the nanocarriers and biological systems should be fully explained. The toxicity of nanocarriers is also a significant issue, and a lot of research groups are actively engaged in designing and manipulating the nanocarriers to mitigate their toxicity. Nanocarriers act drastically when they are reduced to tiny particles. Standard rules are not applied to this “nanoscale” in the same manner as they work at the macro-scale. On the macro scale, the majority of characteristics of the substance predominate over the surface properties. Also at the micro-scale, surface properties tend to predominate. Both forms of properties play a major role in the mesoscale (Medintz et al., 2005; Zaib and Iqbal, 2019). Besides that, the effects of metabolized/modified nanostructures on the biological system are hard to guess. Regulatory agencies are taking steps to test emerging nanocarriers, their properties, and biocompatibility with the host. Though manufacturing nanocarriers from the laboratory to mass production is hard, and the materials applied to produce nanocarriers are costly still we hope this technology will be a promising one for combating certain long-lasting and un-treatable diseases like cancer.

## Conclusion

Nanotechnology has demonstrated numerous benefits for the topical and transdermal delivery of medications. It has already been reported for different medications that topical/transdermal formulations composed of NPs can boost skin penetration, increase treatment effectiveness, target epidermis or follicles, and minimize the adverse events. Besides, enhanced activities have been recorded. Most of these delivery systems can be used for both hydrophilic and lipophilic particles. Further development within the structures, processing processes, and procedures promote the production of new and improved nanocarriers. Combined physical methods and nanocarrier based drug delivery has seen much progress in the field of topical drug delivery. However, future research needs to properly establish a unified system for a range of drugs to be used in nanocarriers and calculate their risk ratio.

## Author Contributions

Y-QY and XY collected the data and drew the figures. X-FW and Y-BF wrote the manuscript. All authors contributed to the article and approved the submitted version.

## Conflict of Interest

The authors declare that the research was conducted in the absence of any commercial or financial relationships that could be construed as a potential conflict of interest.
